# Towards 3D determination of the surface roughness of core–shell microparticles as a routine quality control procedure by scanning electron microscopy

**DOI:** 10.1038/s41598-024-68797-7

**Published:** 2024-08-02

**Authors:** Deniz Hülagü, Charlie Tobias, Radek Dao, Pavel Komarov, Knut Rurack, Vasile-Dan Hodoroaba

**Affiliations:** 1 Division 6.1 Surface and Thin Film Analysis, Federal Institute for Materials Research and Testing (BAM), Unter den Eichen 44-46, 12203 Berlin, Germany; 2 Division 1.9 Chemical and Optical Sensing, Federal Institute for Materials Research and Testing (BAM), Richard-Willstätter-Straße 11, 12489 Berlin, Germany; 3 NenoVision S.R.O., Purkyňova 649/127, 612 00 Brno, Czech Republic

**Keywords:** Core–shell particles, Image analysis, Roughness, Scanning electron microscopy, Atomic force microscopy, Tilting, Batch analysis, Techniques and instrumentation, Characterization and analytical techniques, Imaging techniques, Microscopy, Surface patterning

## Abstract

Recently, we have developed an algorithm to quantitatively evaluate the roughness of spherical microparticles using scanning electron microscopy (SEM) images. The algorithm calculates the root-mean-squared profile roughness (RMS-R_Q_) of a single particle by analyzing the particle’s boundary. The information extracted from a single SEM image yields however only two-dimensional (2D) profile roughness data from the horizontal plane of a particle. The present study offers a practical procedure and the necessary software tools to gain quasi three-dimensional (3D) information from 2D particle contours recorded at different particle inclinations by tilting the sample (stage). This new approach was tested on a set of polystyrene core-iron oxide shell-silica shell particles as few micrometer-sized beads with different (tailored) surface roughness, providing the proof of principle that validates the applicability of the proposed method. SEM images of these particles were analyzed by the latest version of the developed algorithm, which allows to determine the analysis of particles in terms of roughness both within a batch and across the batches as a routine quality control procedure. A separate set of particles has been analyzed by atomic force microscopy (AFM) as a powerful complementary surface analysis technique integrated into SEM, and the roughness results have been compared.

## Introduction

Core–shell (CS) particles are attracting increasing attention in a wide variety of applications from drug delivery and tissue engineering to catalysis, separation, and materials processing, and also include (bio)sensory particles and microenergetic materials^1–8^. In addition to the advantages brought by the combination of organic and inorganic materials in one bead, the designed particles can be endowed with distinctive properties by controlling the size and architecture of the single domains or by changing the core-to-shell ratio^1,6,7,9^. Moreover, to meet the diverse application requirements the functionality of the particles can potentially be increased by modification or tailoring of the surface roughness^1^. A versatile organic/inorganic core–shell structure results from the combination of the benefits of a polystyrene (PS) core and a silica (SiO_2_) shell in a micrometer-sized bead format^10–13^. Due to their rapid reaction kinetics, the requirement of small sample volumes, and their ability to be functionalized with (bio)chemical binders in a straightforward manner, this combination is especially advantageous for the quantitative detection of analytes of interest by single particle-based (bio)analytical methods^14–18^.

A key step in the design of functional particles is to connect morphological features which can be synthetically controlled to final performance parameters. The characterization of the chemistry of the bulk as well as the surface of CS particles is performed by well-established analytical methods^1,5,19–23^. However, their detailed morphological characteristics are usually only qualitatively and subjectively described. Besides size and size distribution, shell thickness, surface area and texture, the characterization of the surface morphology of CS particles is important since the functionality of the particles is strongly influenced by their surface roughness. Scanning electron microscopy (SEM) is probably the most common technique that reveals information on the above-mentioned characteristics of the particles^23,24^. Furthermore, SEM provides data on the chemical composition of the samples when equipped with detectors for Energy Dispersive X-ray-Spectroscopy (EDS)^25–27^.

It was reported that the surface roughness of spherical particles has been characterized either by estimating the average roughness value from the cross-sectional profile of the particle or the root mean square (RMS) deviation of the surface from its average level obtained from SEM micrographs, AFM or optical microscope images^28,29^. The fractal dimension with the divider method, area-perimeter fractional dimension, and Fourier analysis are other methods that are employed by researchers based on SEM images^30–32^. However, these techniques are typically two-dimensional (2D) and used for planar profiles of granular materials. On the other hand, a high-resolution SEM image of an individual spherical particle contains rich information on the surface morphology of the top hemisphere of the particle at the nanometer scale. The number of secondary electrons (SE) that are collected by the detector, generates a significant variation between the grayscale values in the recorded image. However, it is a challenging task to determine quantitatively the overall surface roughness by means of this topographic contrast that is contained in an SEM image. Such an approach needs computational programming to obtain a three-dimensional (3D) reconstruction based on SEM images^33,34^.

Recently, there have been contributions to providing 3D surface structures from 2D SEM micrographs. Tondare^35^ discussed obtaining three-dimensional (3D) particle models by applying structure-from-motion (SfM) algorithms to multiple rotational SEM images of particles using a cylindrical substrate. Töberg and Reithmeier^36^ demonstrated 3D reconstruction of surface structures of gravel particles with diameters ranging from 300 to 1000 µm by applying affine camera models to SEM images. They discussed the limitations and the extend of achievable accuracy. Dong et al.^37^ summarized currently used 3D reconstruction algorithms based on SEM images and proposed a 3D SEM imaging method in their work. These approaches have the potential to become a component of SEM-based particle metrology. However, the available approaches mostly do not work reliable for such fine morphologies in the range of a few nanometer which is the limit of detection of a high-resolution SEM.

In order to characterize the surface roughness, Atomic Force Microscopy (AFM) is usually employed owing to its nanometer lateral resolution and high sensitivity to height measurement in the sub-nanometer range^38,39^. It has to be noted that the AFM typically has limitations to characterize curved surfaces of objects such as micrometer-sized CS particles. Since the AFM tip cannot reach the edge region of the particle, the images obtained by AFM need post-processing by a software to be combined with the particle dimension and the tip size and shape. This usually results in a discrepancy between the reconstructed and the actual shape of an analyzed particle^40^. Similar to SEM-based analysis, to achieve a complete 3D analysis of an object by AFM, computer visualization algorithms are used in combination with advanced geometrical and mathematical approaches^41^.

In our previous work^9,42^, we have presented a robust protocol to quantify roughness of a core–shell microparticle by means of accurate detection of the particle’s contour from SEM and transmission mode in SEM (TSEM, or STEM-in-SEM) images by using a self-developed algorithm. This roughness analysis tool, however, yields only a 2D data set from the recorded SEM image which does not provide the roughness of the complete surface of the particle, but the profile roughness extracted from the horizontal section plane (in other words, from the “belly”) of a particle. A practical way to collect information over a larger area of the surface of a spherical particle is to tilt the sample (the stage), allowing the acquisition of images of the particle in different orientations (tilts) at the same detection angle, which in our study would be symmetrical from the top with an SE InLens detector. From the analysis of such images, quasi-3D surface information can be derived. While, by definition, measurements taken from a (ideally) spherical object under different tilting angles should yield identical results, for synthesized composite objects such an ideal state is hardly reached. Thus, such an analysis approach reveals information about the morphological surface homogeneity of a single core–shell particle, the homogeneity of a particle batch in terms of roughness, and the variation of surface roughness from batch to batch. Here a systematic study is presented by measuring more single core–shell particles and larger surfaces of the single particles compared to our previous work^9^, allowing a more representative data generation with respect to the analyzed area per particle.

This work also presents the latest version of our previously reported software code designed to calculate the particle’s profile roughness with high accuracy and reproducibility, as a robust and semi-automatic procedure based on images recorded by SEM as well as TSEM^9^. The latest version of the software code of Python for fully automatic image analysis is freely available on the GitHub repository Roughness-Analysis-by-Electron-Microscopy^43^, allowing users to apply it to their own particles and images. The latest version used for the calculations in this work is improved with new functionalities and enhanced in the sense that it provides a fully automatic quantitative evaluation of profile roughness of a single particle including automatic thresholding for image segmentation.

Serving as a robust complementary method for analyzing surfaces, AFM has been employed in conjunction with SEM (AFM-in-SEM) to study a distinct set of particles. The findings from AFM-in-SEM have been carefully assessed and compared with those obtained from SEM.

This paper proposes a new approach as a proof of principle, demonstrating the feasibility and potential applicability of the developed fully automatic image analysis tool for quantitative surface roughness determination of spherical beads.

## Materials and methods

### Core–shell microparticles

**Figure 1:**
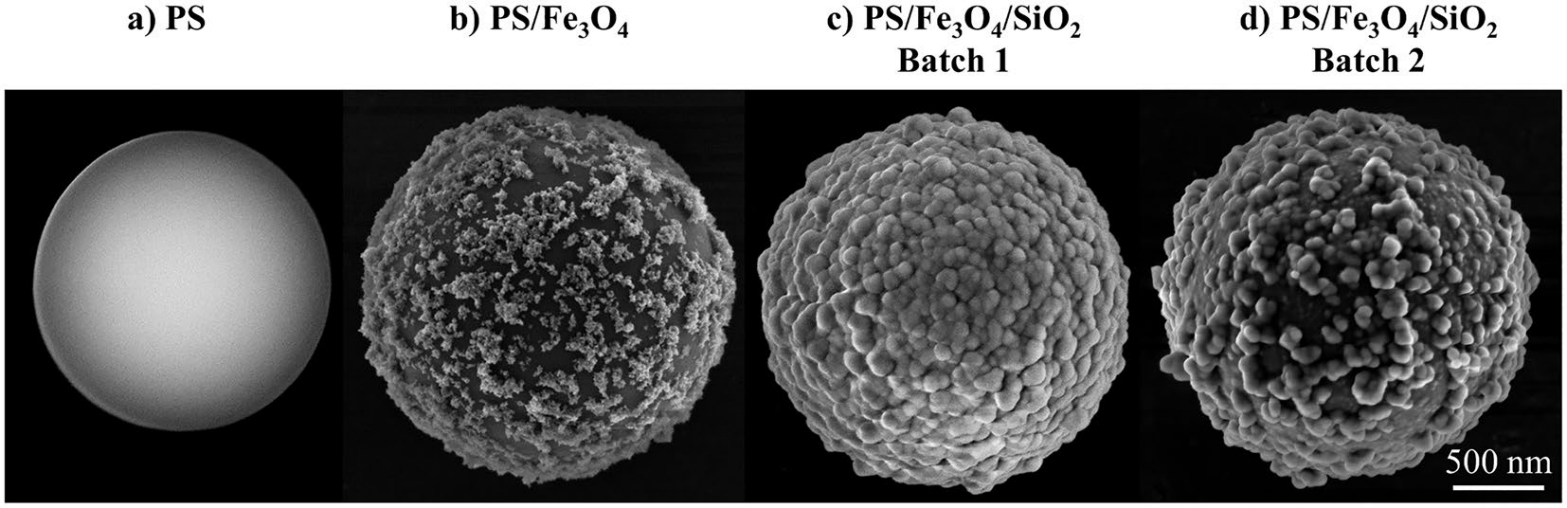
Representative SEM images of the set of particles produced along the synthesis workflow, differing in roughness: (**a**) PS core particle, (b) PS/Fe_3_O_4_ core–shell particle, (**c**) PS/Fe_3_O_4_/SiO_2_ core–shell–shell particle, and (**d**) PS/Fe_3_O_4_/SiO_2_ core–shell–shell particle from another batch than the particle in (**c**).

The investigated particles were produced in our laboratories as previously described in Tobias et al.^17^ (Fig. 1). To represent the smoothest surface condition, highly mono-disperse polystyrene (PS) core particles of an average diameter of d_mean_ = 1.7 ± 0.1 µm were used as a reference sample (Fig. 1a). As previously reported by us^9^, PS core particles were deliberately coated with superparamagnetic iron oxide (Fe_3_O_4_) nanoparticles (d_mean_ = 7 ± 1 nm) to introduce a magnetic functionality, forming a first shell with island-type structured surface on the smooth PS core (Fig. 1b). The resulting PS/Fe_3_O_4_ particles were coated with a second protective silica shell with tunable shell thickness, resulting in PS/Fe_3_O_4_/SiO_2_ beads that are protected against unwanted chemical reactions with the PS core during further functionalization (Fig. 1c,d). The process was mediated by the use of poly(vinylpyrrolidone) (PVP) in the preparation of both the polystyrene and the iron oxide particles.

For definition purposes, “batch” is simply used in this work to describe a quantity of particles produced at one time. The only possible difference from batch to batch should be the time of production since all other production steps remain the same. The main error in production likely arises from the type of stirring employed and the random formation of the silica shell. While the particles will have the same shape, the exact pattern is not reproducible for every single particle.

As these particles were designed specifically for use in (bio)analytical assays, surface structuring is an important feature of the design. The materials used and the details of particle synthesis can be found in the Supporting Information, Sect. 1. Further details on the scattering properties of the particles, their primary chemical functionalization and surface charges as well as their chemical composition and features of the Fe_3_O_4_ nanoparticles can be found in the Supporting Information of our previous work^9^.

### Instrumentation and image acquisition

#### SEM technique

A scanning electron microscope (SEM) of type Zeiss Supra 40 (Zeiss, Oberkochen, Germany) that is part of a laboratory accredited according to ISO 17025 was used in this work. The magnification of the instrument was calibrated on a regular basis according to ISO 16700:2016 Microbeam analysis—Scanning electron microscopy—Guidelines for calibrating image magnification, using dedicated certified reference materials. For the magnification range used in this study an uncertainty of 10% is associated to the distances measured at the nanometer scale.

The SEM is equipped with a high-resolution cathode (Schottky field emitter), an Everhart–Thornley secondary electron (SE) detector and an SE InLens® detector. Detection with the high-resolution, surface-sensitive SE InLens® detector is especially important when the surface morphology of the sample at nanometer scale is of great interest, i.e., as for the purpose of this study.

The particles were suspended in ethanol and ultrasonication was applied for 5 min. The samples were prepared by drop-casting on conventional carbon TEM grids. For the analysis, each grid was placed on the dedicated transmission sample holder, which is described elsewhere^9,27^. To mitigate unwanted effects that might occur due to charging, all SEM images were recorded at a low accelerating voltage of 2 kV from a small working distance of 2.8 mm and quick scan times of frames were applied. No thin conductive coating of the sample was necessary.

Only SEM images of individual particles were recorded with the top-view SE InLens detector at different tilt angles with stepwise tilted sample (stage). First, a single particle was selected from the material batch to be investigated. After all required parameters of the SEM instrument (such as accelerating voltage, working distance, aperture, contrast, and brightness) were set to take high-resolution images, the first image of the particle was recorded without tilting. The same image recording procedure was repeated at 2, 4, 6, 8, and 10 tilt degrees. Following that, the tilting degree was set to 0° again, and 6 more images of the same particle were recorded under identical conditions at the previously applied tilting steps of 0, 2, 4, 6, 8, and 10 degrees.

The tilting option was available only in one direction on the SEM set-up. For some selected particles, the sample stage was rotated by 180° after recording the images at different tilt angles, and the measurements were repeated at identical tilt angles up to 10° as it was carried out before without rotation. Thus, more 2D boundaries, and hence a larger surface of the particle was investigated compared to the particles which were tilted up to 10° without rotation. One of the particles was stepwise tilted up to ± 25° to get a better understanding of variation in roughness value over a broader range of tilt angle.

The recorded SEM images were automatically analyzed with respect to the roughness of the particles by the latest version of the software code self-written in Python. Calculated roughness results are presented either as a function of tilt angle or as the arithmetic mean of the calculated roughness values from each of the two corresponding images taken at the same positive and negative tilt angle. In this way, more images were taken at multiple tilt angles and the reproducibility and representativity of the roughness measurements was evaluated.

The approach proposed in this work permits accessing more particle surface by taking several SEM images from a series of different tilt angles and putting together the information obtained from these images. This provides information about the surface roughness of the investigated particles that goes beyond a single 2D lateral profile. In addition, the data generated with our approach by merging the obtained information from tilted images yields more reliable results than using only one single SEM image. The tilting angle was extended up to a certain degree to prevent larger working distances necessary for the image acquisition. The more the sample (stage) is tilted, the larger working distance is needed in the SEM setup and both the resolution and the pixel size change, so that the results become incomparable.

#### The image processing workflow from SEM

**Figure 2:**
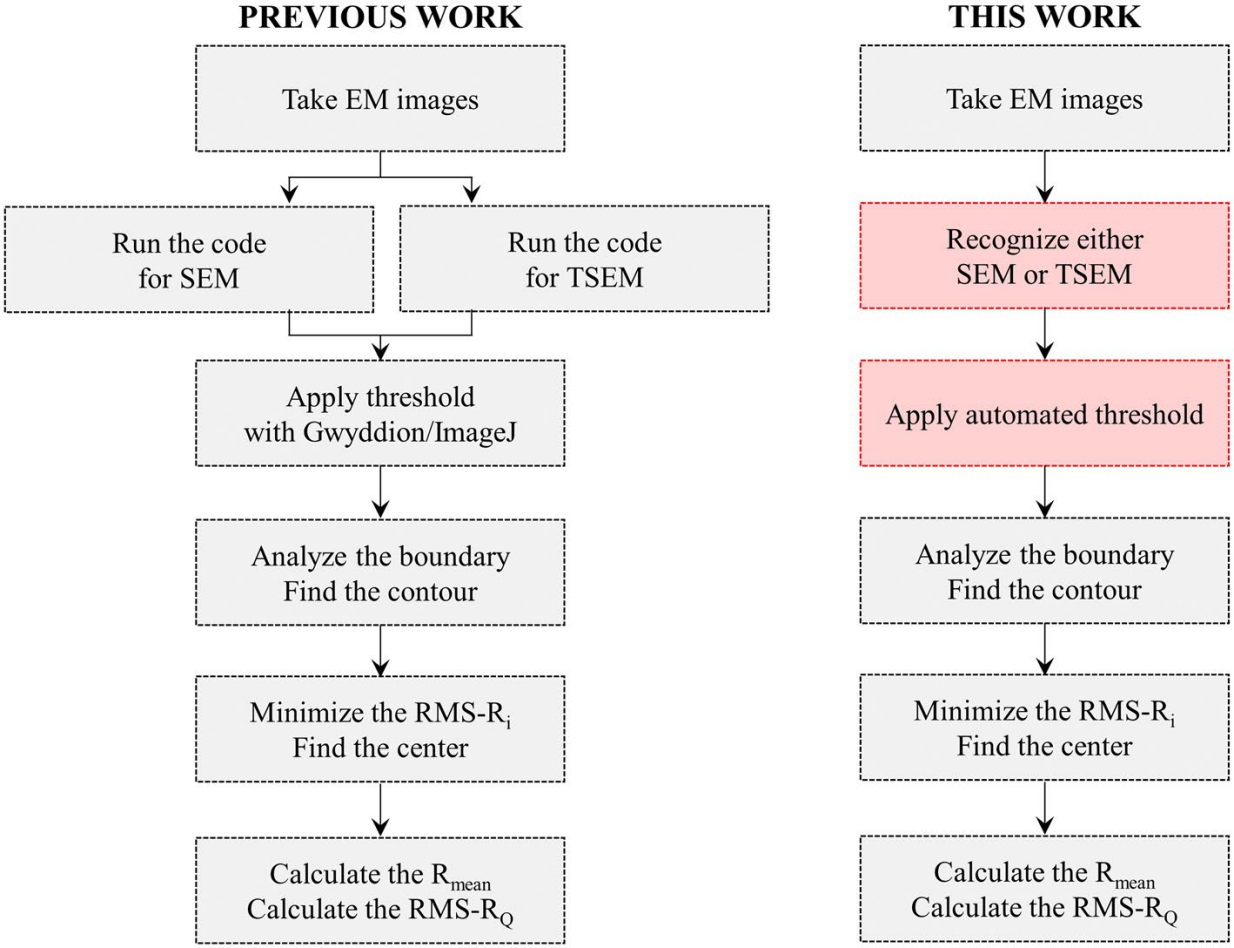
Comparison of the processing sequence of the software code for the image analysis used in our previous work^9,42^ (left) and the latest version used in this work with marked red-framed boxes indicating the updated parts of the code (right).

A comparison of the image-processing sequence of the software code used in our previous work^9,42^ and the latest version used in this work is depicted in Fig. 2. In the previous work, EM images were analyzed by a semi-automatic workflow to obtain the particles’ profile roughness. Two different options of the software code were designed to run separately for SEM or TSEM images. The elements of an SEM image matrix are grayscale values that represent the intensity proportional to the number of secondary electrons (SEs) emitted by the material surface being imaged, or transmitted electrons in the case of TSEM. Electrons that pass through the object appear white on the screen, while those that do not pass through result in black areas on the screen. Recorded EM images (SEM or TSEM) are digital images composed of pixels, following common image data formats. Each pixel is encoded on a grayscale ranging from 0 to 255, where 0 corresponds to black, 255 corresponds to white, and the values in between represent various levels of gray, forming a grayscale image. The latest version analyses the grayscale information contained in each pixel of the obtained electron microscopy images. All analysis steps in coding are based on this principle. According to this information it detects whether the image is an SEM or a TSEM, based on the contrast of the individual mode of electron microscopy. Therefore, it is not required to run two separate software packages for SEM and TSEM, as this novel capability is included in the latest version. It is worthy to note here that the algorithm performs image scanning starting from the top-left corner, moving to the right for each row. This process continues until the image tag information box at the bottom of the image. Otherwise, the pixels within this box, which have different grayscale values than the rest of image, can lead to misleading results when detecting the particle’s contour. However, the algorithm can be further developed to extract the necessary information from the mentioned box.

As the following step, image segmentation (thresholding) is essential to classify pixels of the image as being foreground or background, so that the object of interest can certainly be identified. Thresholding was found to be the most critical step causing significant measurement uncertainties. It was previously performed manually by choosing one of the two different freely available software packages: Gwyddion^44^, which is commonly used for AFM, or ImageJ^45^. The new code applies segmentation automatically to binarize the EM image based on IsoData algorithm, which was originally proposed by Ridler and Calvard in 1978^46^.

**Figure 3:**
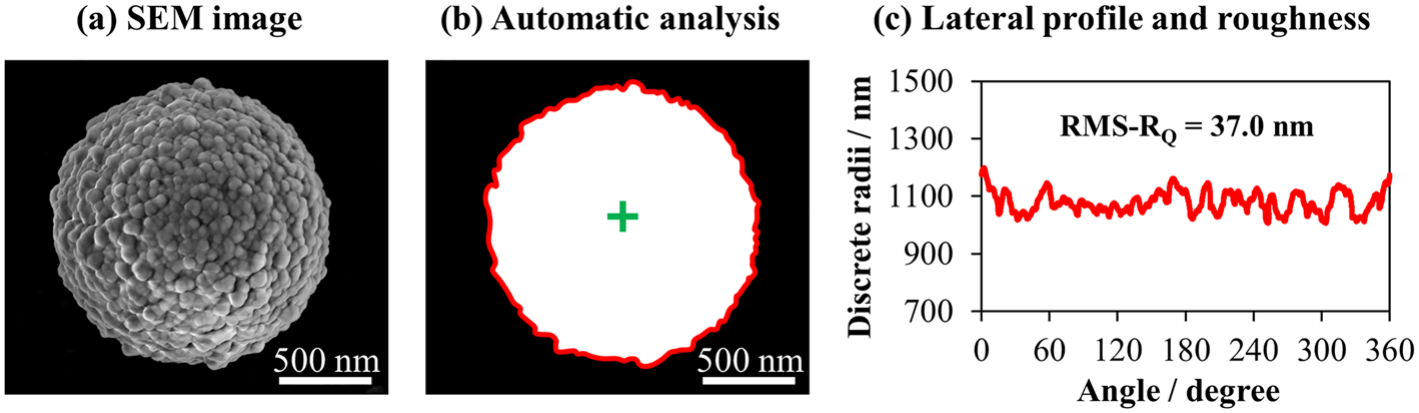
Illustration of the process sequence of the developed image analysis software code: (**a**) an exemplary SEM image, (**b**) segmented SEM image with identified contour (red line) and center of the particle (green plus mark), and (**c**) lateral profile of the particle (red) and the extracted root-mean-squared roughness (RMS-R_Q_).

The next steps in the image processing workflow were the same as in the previous version, see Fig. 3 in case of an SEM image. The software code scans over each pixel of the binary image and identifies all possible boundary points when the pixel changes from black to white, or vice versa. Hence, the complete contour of the particle is identified (red line in Fig. 3b).

To find the center point of the particle, an initial center point is estimated as the average of the identified border points. The distances (d_i_) between this initial center and each identified boundary point are calculated. Then, the software code runs an iteration, and, consequently, the optimized center point (green plus mark in Fig. 3b) is calculated by minimizing the standard deviation (SD) of the distances d_i_ between the center point and each border point. At the end of the iteration, the final distances are calculated as the discrete radii (d_r_). To obtain the lateral profile of the particle, the resulted discrete radii d_r_ are plotted against the respective angle as presented in Fig. 3c. As the final outcome of the software, the root-mean-squared profile roughness (RMS-R_Q_) of the particle projection is calculated as follows:

1 $${\text{RMS-R}}_{\text{Q}}{ = }\sqrt{\frac{1}{{\text{N}}}\sum \limits_{\text{i=0}}^{\text{N}}{{{(}{\text{d}}}_{{\text{r,i}}}-{d}_{avg})}^{2}},$$

where *N* is the number of pixels that define the contour, *i* is the boundary point, *d*_r,i_ is the discrete radius between the optimized center point and the boundary point of *i*, and *d*_*avg*_ is the mean of discrete radii.

#### AFM-in-SEM technique

A separate set of surface roughness measurements was performed with an AFM (LiteScope™, NenoVision) integrated into an SEM (MIRA3 XMU, Tescan), called AFM-in-SEM, at CEITEC Nano in Brno, Czech Republic for correlative in-situ 3D analysis of the four different types of particles.

The combination of AFM and SEM enabled the identification of the spherical particles by SEM and immediate navigation of the AFM tip directly to the surface of the chosen particles with nanometer precision. The total area reachable by the tip was 21 mm × 12 mm offering plenty of particles to be investigated. The topography was measured in the dynamic semi-contact regime.

**Figure 4:**
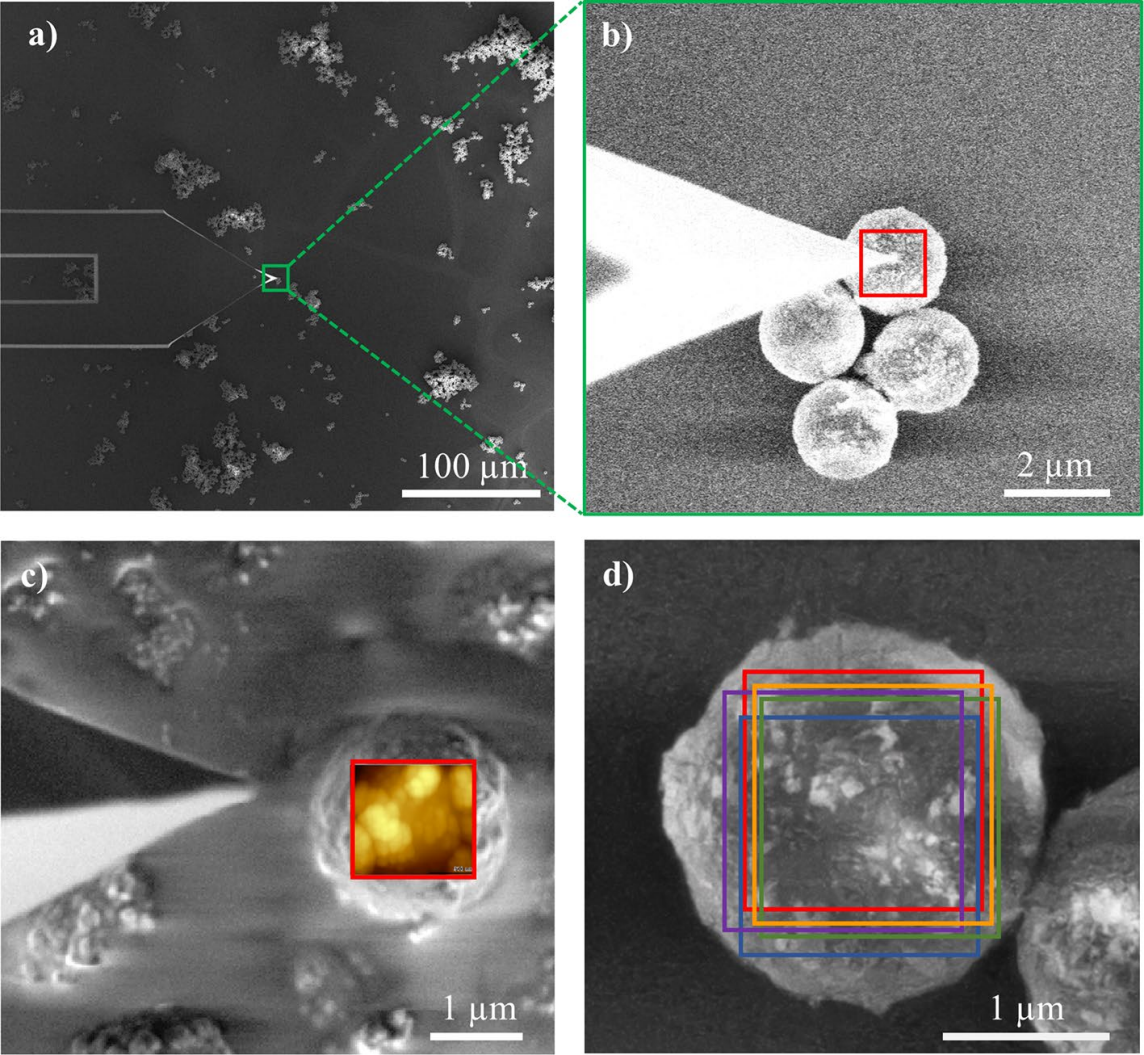
An example illustrating AFM measurements of PS/Fe_3_O_4_ core–shell particles: (**a**) overview of the AFM tip and PS/Fe3O4 core–shell particles, (**b**) higher magnification view of the particles with roughness measurement range of 1.4 µm × 1.4 µm area indicated as a square frame in red, (**c**) post-processed AFM-in-SEM data using Gwyddion software, (**d**) calculation of average roughness from five 1.4 µm × 1.4 µm subsections per image.

Figure 4 presents example AFM measurements conducted on PS/Fe_3_O_4_ core–shell particles. Figure 4a provides an overview of the AFM tip and the particles. Figure 4b shows a higher magnification view of the particles measured with the AFM tip. Post-processing of AFM-in-SEM data was performed using Gwyddion software, which involved subtracting a parabolic background. This process generated a map of vertical displacement within 1.4 µm × 1.4 µm areas, crucial for calculating the RMS surface roughness of each particle, as indicated by the red square box in Fig. 4b,c. The RMS values were computed using statistical tools available in Gwyddion software, which offers moment-based surface roughness determination^44^. AFM measurements were conducted five times on different 1.4 µm × 1.4 µm areas of each image. The average roughness values of the particles were calculated by analyzing these five separate areas (Fig. 4d). Uncertainties in the AFM-in-SEM method were assessed based on these repeated roughness calculations.

## Results and discussion

### Roughness analysis of a batch

For each type of particles, SEM images of several randomly selected individual particles from the same batch were recorded at identical image acquisition conditions, such as accelerating voltage (2 kV), working distance, scan speed, magnification, and pixel size.

**Figure 5:**
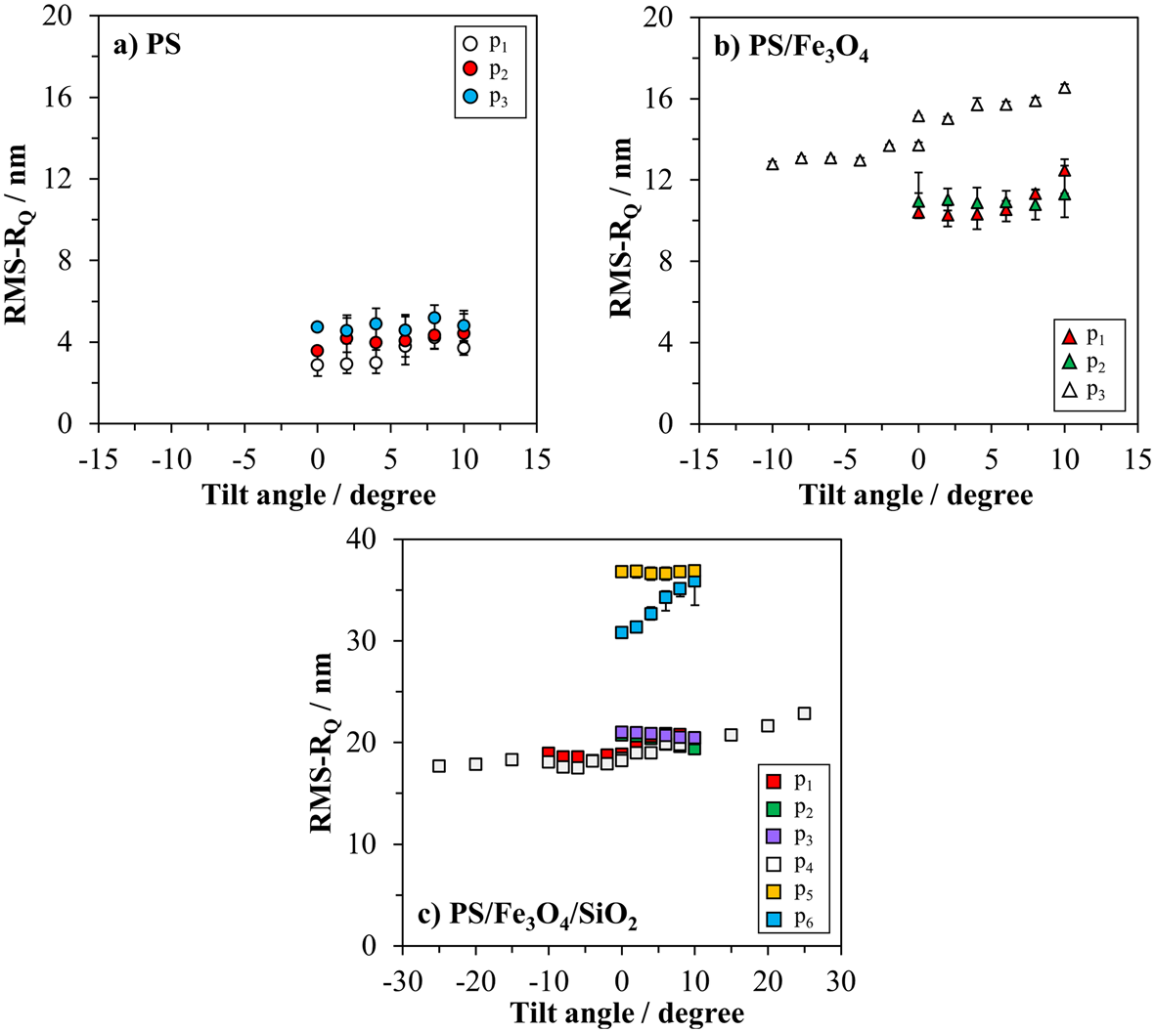
Automatically calculated SEM-based roughness values of (**a**) PS core particles, (**b**) PS/Fe_3_O_4_ core–shell particles, and (**c**) PS/Fe_3_O_4_/SiO_2_ core–shell–shell particles as a function of tilt angle. Images were recorded at 2 kV. Uncertainties are determined from repeated measurements and image analysis (p_x_ = individual particle).

Three particles were randomly selected from the first type of particle, i.e., bare PS particles. The particles were tilted on one side up to 10° in steps of 2°. The set of tilting measurements of the individual particles was repeated twice under identical conditions. The subsequent analysis involved calculating the roughness values from SEM images recorded at each tilt angle with the final output representing the arithmetic mean of the roughness values derived from paired images as plotted in Fig. 5a as a function of the tilt angle. The uncertainties presented in Fig. 5 are determined from the measurements obtained by repeating the image acquisition sequence twice, followed by corresponding image analysis. As expected, the determined roughness values showed no significant differences with tilting for all three particles, constituting the smoothest, spherical reference sample. Practically, the same features but different gray values of decisive pixels were observed on the contour of the particles. The overall average of the calculated roughness values from the images recorded at different tilting degrees were 3.8 ± 1.6 nm for particle p_1_, 4.1 ± 1.3 nm for particle p_2_ and 4.8 ± 1.0 nm for particle p_3_. These results suggest that the particles have almost the same average roughness value, which means an acceptable homogeneity of roughness within the batch.

The same image recording procedure with tilting was performed with three randomly selected individual particles from the second type of particle, i.e., PS/Fe_3_O_4_ core–shell particles. Particle p_1_ and particle p_2_ were tilted on one side up to 10° in increments of 2° and two sets of images were recorded per particle by repeating the imaging process twice under identical conditions. As presented in Fig. 5b, roughness values of particle p_2_ remained the same within the 10° tilt angle range. Similarly, the roughness values of particle p_1_ remained the same up to 6°, however, slightly increased when the degree of tilting was ≥ 8°, with various prominent features on the contour of the particle becoming visible in these projections. SEM images of the particle p_3_ were recorded with the same regime. To probe a larger part of the particle’s surface, particle p_3_ was horizontally rotated by 180° and the same set of tilting stepwise up to 10° was repeated on the opposite side. This means that particle p_3_ was tilted twice on opposite sides spanning a tilt angle range of 10°. This ensured a comprehensive examination of the particle's surface under more orientations. The roughness values of particle p_3_ slightly increased from 0° to 10°, however, remained unchanged when the particle was tilted after 180° horizontal rotation (tilt angles from 0° to − 10° in Fig. 5b). This indicates that the large features of the shell material located on the region where we tilt through + 10° became more and more visible and increased the obtained roughness value. Particles p_1_ and p_2_ showed very similar average roughness values (11.2 ± 1.5 nm and 11.5 ± 1.5 nm, respectively) which were slightly lower than for particle p_3_ (14.7 ± 2.0 nm). It can be concluded that the batch of PS/Fe_3_O_4_ core–shell particles appears to be homogenous in terms of roughness, with the remark that the analysis exercise was carried out on only a few particles for proof-of-principle purpose. Moreover, the calculated roughness values of PS/Fe_3_O_4_ core–shell particles were, as expected, significantly higher than those of bare PS particles, which makes it possible to clearly distinguish these two types of particles regarding their roughness by SEM.

Similar to previous types of particles, tilting measurements and following image analysis of the PS/Fe_3_O_4_/SiO_2_ core–shell–shell particles were also repeated twice in order to determine uncertainties from the repeatability. Figure 5c shows calculated roughness values of six individual particles selected randomly from the type of PS/Fe_3_O_4_/SiO_2_ core–shell–shell particles as a function of tilt angle. It is clearly seen that this type of particles, even if analyzed in a reduced number, showed the highest roughness among the investigated samples. Both particles p_5_ and p_6_ were tilted from 0° to 10° without horizontal rotation. Although the roughness values of p_5_ remained constant at 36.6 ± 0.7 nm over the tilt range, an increment in roughness of p_6_ was observed from 30.8 ± 0.4 to 35.1 ± 1.7 nm, which equals to a variation of 10%. In the case of p_6_, since some of the prominent features on the surface of the particle became more visible when the sample was tilted up to 10°, the calculated roughness value was accordingly larger. On the other hand, particles p_2_ and p_3_ were tilted up to 10° without rotation and showed almost the same average roughness values as 20.0 ± 1.0 and 20.8 ± 0.7 nm, respectively. Particle p_1_ was tilted ± 10° with horizontal rotation, and the calculated roughness values showed an *s*-like trend when plotted as a function of tilt angle. This *s*-like tendency is explained by the fact that the prominent features on the particle’s surface gradually became more visible and gradually disappeared, as the particle was stepwise tilted in both directions, respectively. Nevertheless, the average roughness value of particle p_1_ determined as 19.5 ± 1.4 nm was not noticeably different than those of particles p_2_ and p_3_.

Unlike previous measurements, one of the particles in this set (particle p_4_) was tilted up to ± 25°, and a similar trend was observed from the roughness values of this particle p_4_ having an average of 20.2 ± 2.7 nm. Since this particle was examined over a larger tilt angle range, the variation in the roughness value was higher (13%) than those of other particles having almost the same average roughness values among this particle type such as p_1_, p_2_, and p_3_.The trend of the roughness variation of particle p_4_ is explained in detail in the Supporting Information Sect. 2.1. in dependence on the obtained lateral profiles.

### Batch-to-batch roughness analysis

To evaluate the batch-to-batch homogeneity of the surface roughness values, another batch of PS/Fe_3_O_4_/SiO_2_ core–shell–shell particles (batch 2) was investigated and the obtained results were compared with the data of the previous batch of the same particle type (PS/Fe_3_O_4_/SiO_2_ core–shell–shell particles, batch 1).

**Figure 6:**
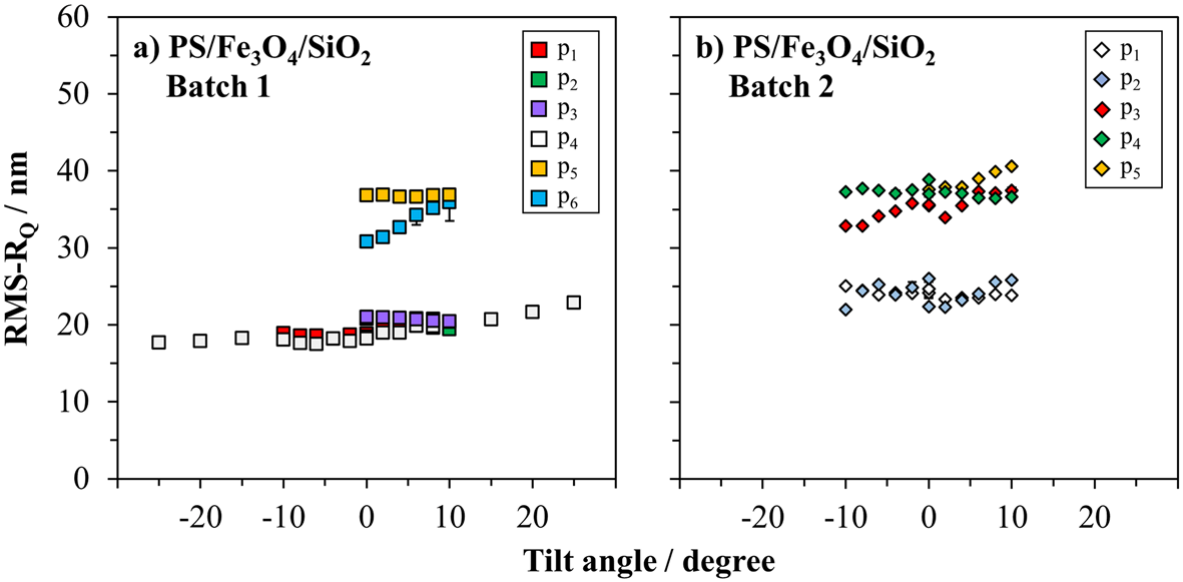
SEM-based roughness values of PS/Fe_3_O_4_/SiO_2_ core–shell–shell particles (**a**) from the first batch and (**b**) from the second batch, as a function of tilt angle. Images were recorded at 2 kV. Uncertainties are determined from repeated measurements and image analysis (p_x_ = individual particle).

Five arbitrarily selected particles were investigated from the second batch of PS/Fe_3_O_4_/SiO_2_ core–shell–shell particles. SEM images of these particles were recorded under identical measurement conditions, as previously performed for the particles investigated in Sect. 3.1., with measurements and image analysis repeated twice. Figure 6b presents the roughness values of the particles automatically calculated by the software code. For ease of comparison, previously discussed data of PS/Fe_3_O_4_/SiO_2_ core–shell–shell particles from the first batch are shown in Fig. 6a.

Particles p_1_ and p_2_, having average roughness of 24.2 ± 0.9 nm and 24.0 ± 1.7 nm, respectively, showed lower roughness values than the other particles investigated from this batch, as depicted in Fig. 6b. The roughness values of particles p_1_ and p_2_ did not vary significantly when the samples were tilted up to ± 10°. In case of both particles p_3_ and p_5_, a slight increase of roughness (7% and 4%. respectively) was observed from − 10° to + 10° having average roughness values of 35.2 ± 2.3 nm and 39.1 ± 1.6 nm, respectively . The same effect was observed for the particle p_6_ from the first batch. Contrary, a slight decrease of the roughness values of particle p_4_ was observed (from 38.9 ± 0.1 nm to 37.3 ± 0.2 nm) in the second batch when the sample was tilted up to 10°. Likewise, this effect was obtained when some of the prominent features on the particle surface at 10° became less visible when the particle was tilted. Moreover, as it is clearly visible from Fig. 1c,d, the core–shell–shell particles are never perfectly isotropic, in fact they can hardly become isotropic because the iron oxide layer is intentionally designed (and constructed) as an “islandly layer”.

Upon Fig. 6a,b, it appears that the results may be grouped into two roughness classes. However, to reach this conclusion it is essential to perform a statistical evaluation on a sufficiently large number of particles and a systematic error analysis to set up a corresponding uncertainty budget, but this task is kept deliberately out of the scope of this paper, with the declared focus to demonstrate the proof-of-principle applicability of the proposed analytical approach and related tools.

Additionally, considering inevitable variations occurring during the production of different batches, the roughness values of particles selected from both productions, batch 1 and 2, were found to fall within the similar roughness range as 20 to 40 nm. The values bolded in Table 1 represent the calculated average roughness values of the investigated particles by SEM. These results show that different batches can also be evaluated quantitatively with the developed image analysis tool and this procedure can be used as a routine quality control system for the reproducibility of the results.

**Table 1:** Overview of the average root-mean-squared profile roughness (RMS-R_Q_) values based on the SEM images of tilted particles (values bolded) and based on the AFM-in-SEM images (values not bolded). SEM-based uncertainties are determined from repeated measurements and corresponding image analysis conducted twice. AFM-in-SEM-based average roughness values and uncertainties are calculated from five different subsections (1.4 µm × 1.4 µm) per image (px = individual particle index).

Particle number	RMS-R_Q_ (nm)			
	PS	PS/Fe_3_O_4_	PS/Fe_3_O_4_/SiO_2_ (batch 1)	PS/Fe_3_O_4_/SiO_2_ (batch 2)
p_1_	**3.8 ± 1.6**	**11.2 ± 1.5**	**19.5 ± 1.4**	**24.2 ± 0.9**
p_2_	**4.1 ± 1.3**	**11.5 ± 1.5**	**20.0 ± 1.0**	**24.0 ± 1.7**
p_3_	**4.8 ± 1.0**	**14.7 ± 2.0**	**20.8 ± 0.7**	**35.2 ± 2.3**
p_4_	4.9 ± 0.5	8.6 ± 0.7	**20.2 ± 2.7**	**37.6 ± 1.4**
p_5_	4.2 ± 0.4	10.7 ± 0.5	**36.6 ± 0.7**	**39.1 ± 1.6**
p_6_	4.1 ± 0.4	13.9 ± 0.4	**33.8 ± 3.4**	28.8 ± 4.9
p_7_	–	14.7 ± 0.7	18.8 ± 2.6	32.3 ± 5.5
p_8_	–	–	22.2 ± 3.2	48.6 ± 8.3
p_9_	–	–	23.7 ± 4.0	12.6 ± 2.1
p_10_	–	–	15.4 ± 3.8	35.5 ± 6.0

### Roughness analysis by AFM-in SEM

**Figure 7:**
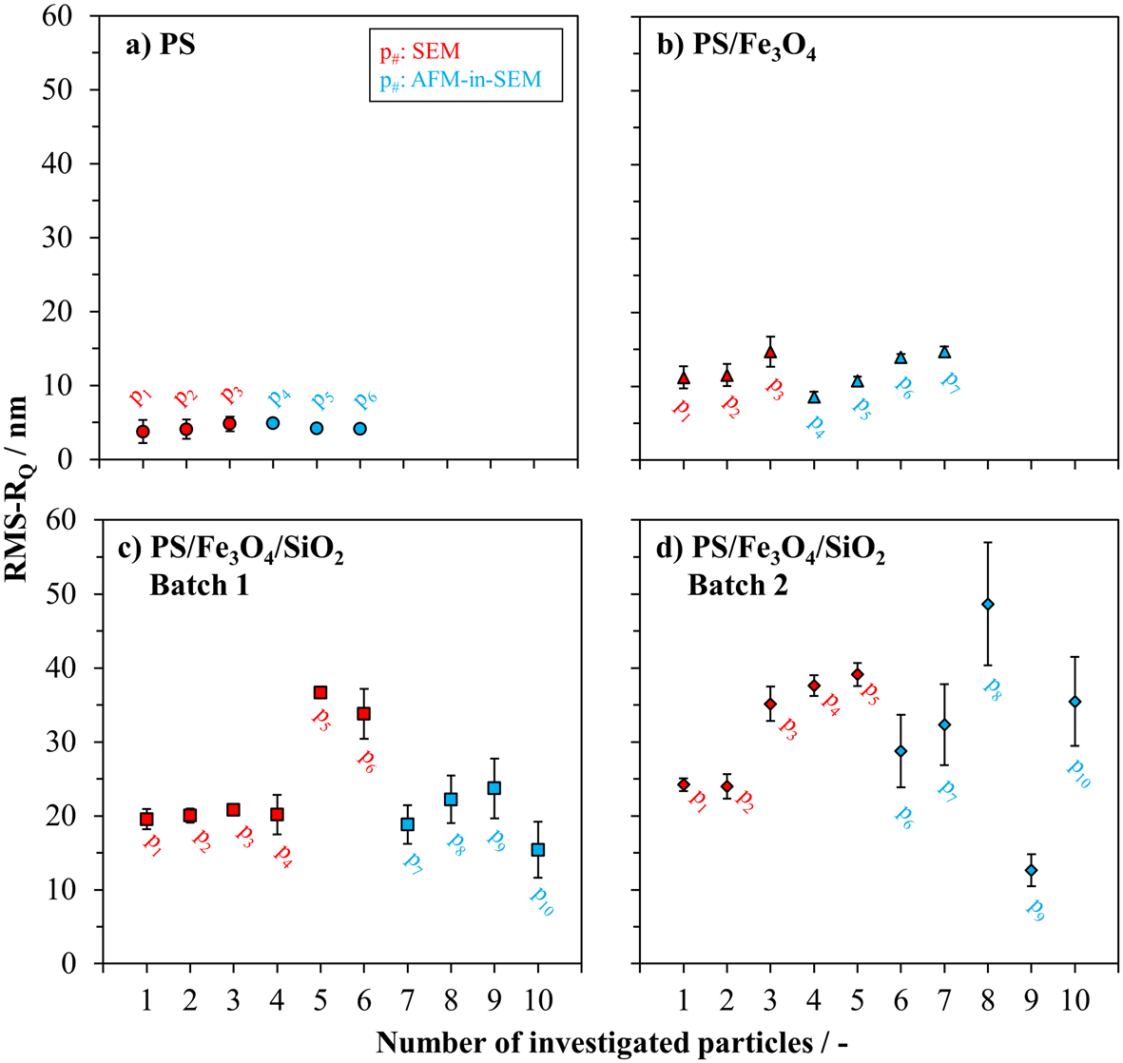
Comparison of roughness values of core–shell–shell particles: (**a**) PS core particles, (**b**) PS/Fe_3_O_4_ core–shell particles, (**c**) PS/Fe_3_O_4_/SiO_2_ core–shell–shell particles, and (**d**) PS/Fe_3_O_4_/SiO_2_ core–shell–shell particles from another batch than the particles in (**c**). The average root-mean-squared profile roughness (RMS-R_Q_) values based on the SEM images of tilted particles are colored in red, the roughness values of the particles calculated from AFM-in-SEM images are colored in blue. Uncertainties are determined from repeated measurements and image analysis (p_x_ = individual particle).

A separate set of measurements were conducted with an AFM-in-SEM as described in “AFM-in-SEM technique” section. The particles measured with AFM-in-SEM technique are different from those measured with SEM technique, but both sets originate from the same production batch. Figure 7 presents roughness values of multiple particles randomly selected from the investigated particle production calculated from the AFM-in-SEM images and the results are compared with the previously calculated roughness values from the SEM images using the automated image analysis tool as listed in Table 1.

Due to the strong charging effect, only three individual particles were measured from the bare PS particles and the calculated roughness values were plotted in Fig. 7a. In the roughness measurements, automatically calculated by the software code from the SEM images recorded at BAM, values of 3.8 ± 1.6, 4.1 ± 1.3, and 4.8 ± 1.0 nm were obtained for three different particles of p_1_, p_2_, and p_3_. The calculations from AFM images fell in the expected range of roughness and resulted in 4.9 ± 0.5, 4.2 ± 0.4, and 4.1 ± 0.4 nm for the particles of p_4_, p_5_, and p_6_ as listed in Table 1, which demonstrates excellent agreement with the independent SEM results. This level of agreement suggests that both sets of measurements are consistent even though factors such as variations in experimental conditions, measurement techniques, or inherent particle differences, and, last but not least, considering only a few particles, may contribute to the calculated results. Furthermore, this good agreement within the uncertainty ranges, which are obtained from repeated measurements and image analysis, underscores the robustness of the results and strengthens confidence in the reliability of both sets of calculations. These results also suggest that the proposed method in this work for determining particle profile roughness is accurate and this shall also be seen as a validation of the results obtained. Furthermore, it was found that by repeating measurements, the charging effects was insignificant.

The surface roughness measurements of four randomly selected individual PS/Fe_3_O_4_ core–shell particles yielded values of 8.6 ± 0.7, 10.7 ± 0.5, 13.9 ± 0.4, and 14.7 ± 0.7 nm as plotted in Fig. 7b (from p4 to p7). As expected, PS/Fe_3_O_4_ core–shell particles showed significantly higher surface roughness values than those of bare PS particles. Observed variations of roughness values between two sets of measurements conducted with SEM and AFM-in-SEM were in good agreement. It is noteworthy that the roughness value of particle p_4_ calculated from the images recorded by AFM-in-SEM technique (8.6 ± 0.7 nm) was slightly lower than the values obtained from the SEM images calculated with the automated image analysis tool. Although this (small) set of particles were found to be quite homogenous in term of surface roughness depending on the obtained results from SEM images, there might be still potential variation sources, such as instrumentation or data post processing. However, the discrepancy of the results obtained with two different measurement techniques and calculation approaches was in acceptable range.

Figure 7c shows calculated roughness values of four individual particles selected randomly from the type of PS/Fe_3_O_4_/SiO_2_ core–shell–shell particles from the first batch (Batch 1). It was previously determined from the SEM images that this type of particles showed the highest roughness among the investigated samples. The calculated roughness values from AFM-in-SEM technique were 18.8 ± 2.6, 22.2 ± 3.2, 23.7 ± 4.0, and 15.4 ± 3.8 nm for particles p_7_, p_8_, p_9_, and p_10_, respectively.

Roughness values of p_7_, p_8_, and p_9_ were found within the roughness range of particles p_1_, p_2_, p_3_, and p_4_ determined by SEM technique. However, the roughness value of p_10_ was positioned slightly lower than the other investigated particles of this batch. This might be due to the slight image deformation at the curved corners of the selected areas on the spherical core–shell–shell particles, which is a well-known limitation of the AFM technique when employed to such highly curved surfaces^47^.

From the second batch (Batch 2) of the same particle type of PS/Fe_3_O_4_/SiO_2_ core–shell–shell particles, images of five individual particles were recorded by AFM-in-SEM (from p_6_ to p_10_) as shown in Fig. 7d. Although the roughness values determined by AFM-in-SEM for this set of particles were considerably spread, the roughness values of particle p_6_, p_7_, and p_10_ (28.8 ± 4.9, 32.3 ± 5.5, and 35.5 ± 6.0 nm, respectively) were located within the roughness range of all particles from p_1_ to p_5_ investigated by SEM technique.

The roughness of particle p8 was the highest with a value of 48.6 ± 8.3 nm. This could be assigned to the huge features on the outer shell of the particle. On the other hand, the roughness of the particle p_9_ was calculated to be 12.6 ± 2.1 nm, as the lowest roughness value among the measured particles of this type, due to the visible damaged surface of the particle. This nature of the particles showed the influence of selecting misshaped or damaged particles’ surfaces to determine the roughness and contributed to the observed discrepancies.

**Figure 8:**
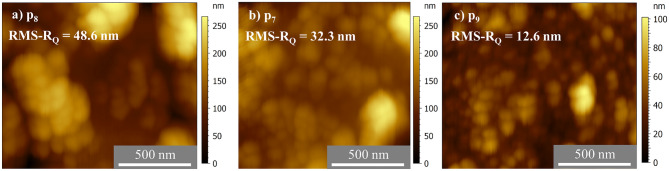
Area profiles of the AFM images of PS/Fe_3_O_4_/SiO_2_ core–shell–shell particles from batch 2 (**a**) particle p_8_, (**b**) particle p_7_, and (**c**) particle p_9_ after subtraction of a parabolic fit.

For a better understanding of the surface nature of the measured particles, a comparison of the subtracted parabolic fit extracted areas of the measured particles p_7_, p_8_, and p_9_ are compared in Fig. 8. In Fig. 8a, large features were observed on the outer shell of the particle p_8_ and black voids were observed in Fig. 8c indicating that the shell did not fully encompass the surface of particle p_9_. The surface of particle p_7_, in comparison to the other two particles p_8_ and p_9_, appeared to be more uniformly coated with the shell material (Fig. 8b). This comparison addresses the role of selecting misshaped or damaged particles to calculate the surface roughness.

## Conclusions and outlook

The roughness of CS microparticles is crucial for their functionality, but measuring it quantitatively is challenging due to the diverse architectures at the nanometer scale, especially when the silica shell is formed. Existing methods using electron microscopy images to determine surface roughness vary significantly. The main purpose of this work is to introduce a generalized, robust, and reproducible method for accurately determining intra- and inter-batch roughness of nonplanar and architecturally complex specimens like CS particles from electron microscopy images. The proposed concept aims to analyze not just a single particle, but to provide a representative assessment of a batch, by offering guidelines for tilted SEM image acquisition and fully automated image analysis. An automated image collection (even if not 100%) could be also envisaged to be possible at modern instruments or in the near future.

A set of three types of core–shell microparticles with various degrees of roughness were synthesized in our laboratories. The surface roughness of these particles was characterized with a high-resolution SEM. To examine the surface from different perspectives and to gain a comprehensive understanding of the particle’s surface, SEM images were recorded at different tilting degrees. For each SEM image, roughness values were calculated to quantify the irregularities and variations in the particle’s surface. This approach allowed for a consolidated representation of the particle’s surface texture, accounting for the variations observed at different tilting angles. The utilization of multiple tilting degrees in SEM imaging provided a more comprehensive view of the particle’s surface characteristics and demonstrated that this can lead to enriched information (more than 2D) on a particle’s surface. Additionally, we have introduced in this work the latest version of our self-developed, fully automated software code to determine root-mean-squared profile roughness (RMS-R_Q_) of the particles. The combination of systematic tilting, SEM imaging, and computed roughness calculation offers a robust methodology for particle surface analysis. This approach enhances the understanding of how surface roughness evolves with tilt, contributing to a nuanced characterization of the particles’ surface.

Bare PS core particles were found to be quasi-3D homogeneous in terms of roughness when the samples were tilted for up to 10°. PS/Fe_3_O_4_ core–shell particles were determined as being homogenous in their average surface roughness within a batch. PS/Fe_3_O_4_ core–shell particles showed significantly higher average surface roughness values than those of PS particles. A slight variation in roughness values of some of the PS/Fe_3_O_4_ core–shell particles was observed when tilted within ± 10°. The third type of particles, PS/Fe_3_O_4_/SiO_2_ core–shell–shell particles, showed the highest average roughness values among the investigated particle types. Furthermore, two different batches of this type of particles showed the largest roughness range.

SEM-based roughness calculations were performed using the fluctuations along the lateral profile of the particle, while AFM-based calculations were performed from the area of the particle. This offers a complementary approach to the results, which cannot be directly compared quantitatively, but whose comparisons make sense in a relative way (e.g., tendencies and variations of roughness values).

Roughness values obtained from the AFM-in-SEM measurements were consistent with those obtained from the SEM images for bare PS particles, PS/Fe_3_O_4_ core–shell particles, and the first batch of PS/Fe_3_O_4_/SiO_2_ core–shell–shell particles. These results indicate that the analysis technique proposed in this work, combination of tilted imaging with SEM and automated image analysis tool, is accurate, even if applied on a reduced number of particles, in a proof-of-principle approach. Only for the second batch of PS/Fe_3_O_4_/SiO_2_ core–shell–shell particles, lower and higher roughness values were obtained than those were calculated from the SEM images. This could be due to the uncertainty of post-processing of recorded AFM images. Several other factors could also contribute to the observed differences in roughness measurements between two measurement techniques: instrument calibration, measurement resolution, sensitivity to sample properties, and variations in the measurement scale, such as selected scanning area or step size. The presence of significant measurement uncertainties suggests that employing superior measurement statistics on a large number of particles will lead to a more accurate determination of the overall uncertainty budgets. This, in turn, will facilitate a more reliable classification of the particles. This systematic measurement task is deliberately beyond the scope of the current work which is aimed to demonstrate the basic applicability of the new measurement approach. The image acquisition by tilting and rotation without sacrificing the main aim of recording high resolution SEM images is tedious and time-consuming due to focus adjustment, ultra-small size of the magnetic iron oxide nanoparticles, the nonconductive bulk polymeric core, and yet not automatized sample preparation. In contrast, fully automatic data generation and image analysis methodology is consistent, fast and user independent. The data obtained from high-resolution SEM images by tilting as proposed in this paper leads to a more informative, yet accurate quantification of roughness than previously achieved.

As a recommendation for future work, considering a realistically acceptable laboratory time, 10 samples per batch could be considered sufficient to offer statistically meaningful results. To provide precise information for better understanding, capturing SEM images of a single PS particle at 6 tilt angles (0, 2, 4, 6, 8, and 10 tilt degrees) took approximately 2 h. The automatic analysis of these images for instance took only few minutes. If the image’s focus, brightness, and contrast values are in perfect condition for analysis, the analysis can even take only seconds.

One potential limitation of the method proposed in this work is the difficulty in analyzing particles that are adjacent to each other. The fully automated image analysis method presented here is not developed for particles that are stuck together. In future studies, the algorithm could be modified in this direction. However, the surfaces of adhered particles are often damaged, and their outer shells broken, making it impossible to obtain the true roughness value and compromising the accuracy of the results.

The selection of a maximum of ± 25 tilt degrees for SEM observations was based on balancing the need for achieving a comprehensive understanding of the particles’ surface and the practical limitations of our SEM setup. However, tilting up to ± 10° was found to provide a sufficient range to obtain representative roughness values for investigated spherical, rather isotropical particles. Based on these results, the following recommendations can be made for future work. Since the proposed tilting SEM image acquisition is laborious and time-consuming, SEM images can be obtained at larger intervals, for instance with 5 tilt degrees, including 180° rotation. By doing so, fewer SEM images of an individual particle will be recorded than those of presented in this work for some particles, and more particles from a batch can be examined within the same timeframe, allowing a fast roughness calculation using the fully automated image analysis tool presented in this work. The possibility to use an eucentric stage, i.e. with maintenance of focus after stage tilt, a tilting range from e.g. − 45° to + 45° (with 15° steps including 180° rotation), can significantly reduce SEM measurement time. It should be noted that even when the eucentric stage tilt is available, the procedures must be tested to ensure their reliable applicability. The stability and precision of the stage movement as well as of the electron beam after extended working periods, are also essential to enable full automation in the collection of image series at different tilt angles. Depending on the freedom to tilt the stage in the respective microscope, even higher tilt degrees with more points shall be considered. This procedure would serve as a guideline for future work and strike a balance between complexity of image acquisition and representativeness of roughness.

### Supplementary Information

Supplementary Information.

## Data Availability

The datasets generated and/or analyzed during the current study (and its Supplementary Information files) are available in the Zenodo repository, (10.5281/zenodo.11108725).
