# Supplementary material for: Towards 3D determination of the surface roughness of core–shell microparticles as a routine quality control procedure by scanning electron microscopy
Source: Sci Rep. 2024 Aug 2;14:17936. doi: 10.1038/s41598-024-68797-7 (PMC11297195; doi:10.1038/s41598-024-68797-7)
Supplement: Supplementary file 1 — Supplementary Information. [file 41598_2024_68797_MOESM1_ESM.docx]

Supporting Information

Towards 3D determination of the surface roughness of core-shell microparticles as a routine quality control procedure by scanning electron microscopy

Deniz Hülagü^a,*^, Charlie Tobias^b^, Radek Dao^c^, Pavel Komarov^c^, Knut Rurack^b^, Vasile-Dan Hodoroaba^a,*^

# Material and methods

## Materials

Poly(vinylpyrrolidone) (PVP10 with an average molecular weight of 10 kD, Sigma), styrene (ReagentPlus, <99%, Sigma), basic alumina (Al_2_O_3_, Brockmann I, Acros) and azo-bis-cyanovaleric acid (ACVA, MP Biomedicals) were used for the PVP-coated polystyrene core synthesis. Tetraethoxyorthosilicate (TEOS, <99%, Merck) was used for the silica coating. (3-Aminopropyl)triethoxysilane (APTES, 99%, Aldrich) was used for amino-functionalization. Ethanol (abs. 99% and96%, ChemSolute) and water of MilliQ grade (BAM) were used as solvents and for washing.

## Particle Synthesis

### Polystyrene core synthesis

PS particles were synthesized by dispersion polymerization as previously reported by us^1^. The polymerization was carried out in a hybridization oven. A solution of 105 mg of ACVA in 10 mL methanol was prepared in an argon atmosphere. In glass vials, 170 mg PVP10 were dissolved in 10 mL ethanol, before adding 1 mL of styrene, previously filtered through basic aluminum oxide. The mixture was flushed with argon for 30 min. The reaction was started by adding 0.5 mL of the ACVA solution and then left stirring overnight at 70 °C under an argon atmosphere. Afterwards, the particles were centrifuged, washed multiple times with water and ethanol, before drying at room temperature.

### Coating of PS cores with iron oxide nanoparticles, PS/Fe_3_O_4_

First, superparamagnetic iron oxide nanoparticles were synthesized. In a round bottom flask, 0.338 g of FeCl_3_ × 6 H_2_O and 0.172 g FeCl_2_ × 4 H_2_O were dissolved in 100 mL Milli-Q water. The solution was flushed with argon for 20 min before adding dropwise a solution of 4 g PVP10 in 58 ml NH_3_ solution (16%). The reaction was stirred for 1.5 h with a mechanical stirrer at 150 rpm. The particles were washed multiple times with water via magnetic separation and subsequently stored in a refrigerator in a concentration of ca. 3% (w/v) in Milli-Q water.

To coat the polymer cores with a superparamagnetic nanoparticle layer, a solution of 60 mg PS and 2 mL Fe_3_O_4_ particles (~ 3% in water) was prepared in 30 mL Milli-Q water in Falcon tubes. Coating was carried out after placing the tubes on a rotator plate at 40 rpm during 1.5 h. Afterwards, the particles were washed twice with water and once with ethanol, before drying.

### Coating of PS/Fe_3_O_4_ particles with silica shell, PS/Fe_3_O_4_/SiO_2_

First, 60 mg of PS/Fe_3_O_4_ particles were dispersed in 30 mL ethanol and 1 mL Milli-Q water. Then, while stirring with a mechanical stirrer at 150 rpm, 555 μL NH_3_ solution (32%) were added, followed by the dropwise addition of 555 μL TEOS. The mixture was stirred overnight at 38 °C, then washed with water and ethanol multiple times and dried at room temperature.

# Results and Discussion

## Tilting experiments

The effect of tilting on SEM images and lateral profiles obtained at different tilt angles are discussed in this Section with an exemplary particle. SEM images of the single PS/Fe_3_O_4_/SiO_2_ particle p_4_ from the first batch were recorded at 0, 2, 4, 6, 8, 10, 15, 20, and 25 degrees of tilt. Then the sample holder was rotated 180° and the measurements were repeated with the identical tilt steps up to 25 degrees as it was done before without rotation, which provided imaging at ±25 degrees. For the sake of simplicity, only six SEM images taken at tilt conditions of 0, 10, and 25 degrees with and without rotation are presented in **Figure S1**. The lateral profiles automatically extracted from this one series of images with the developed image analysis software code are depicted in **Figure S2** (Therefore, results are presented without standard deviation).

It was clearly seen from SEM images that the second, outer silica shell creates features which look like tiny spheres on the surface of the particle being almost evenly distributed all over the surface. However, some features develop larger and become more prominent, due to the design which influence the surface roughness significantly. When the particle contour shown in the green ellipse number 1 in **Figure S1**a is considered, some features became more visible when the sample was tilted to 10 degrees as seen in **Figure S1**b, and became even larger at 25 degrees tilting as seen in **Figure S1**c. This means that when the sample holder is tilted, some prominent features that cannot be properly caught only from a single projection became visible in this way. On the other hand, when the sample holder was rotated to 180° and then stepwise tilted again from 0 to 25 degrees, the features within the same contour region became less visible but the change was not as pronounced as in the case of tilt without rotation.

After processing the SEM images, lateral profiles of the contour of the particle were obtained from the binarized images. **Figure S2**a compares the lateral profiles extracted from the SEM images taken at 0° and 180° rotation of the sample holder without tilting and the calculated root-mean-squared roughness (RMS-R_Q_) values under these two imaging conditions. The extracted profiles were in a good agreement and calculated roughness values from both measurements were the same (18 nm) indicating a high measurement reproducibility.

**Figure S2**b compares directly the lateral profiles extracted from all six SEM images shown in **Figure S1**. The effect of tilting is clearly visible from the variations of the lateral profiles along the contour of the particle. For instance, the features on the contour of the particle seen inside the area shown by the green ellipse labelled 1 became larger and larger when the particle was tilted to 10° and 25°, respectively. As a result, the distances obtained from the lateral profiles in this area are increased, and, consequently, the calculated roughness value of the particle was increased from 18 nm to 20 nm at 10° tilt and to 23 nm at 25° tilt. On the other hand, tilting the particle up to 25° after rotation did not affect the calculated roughness and the values remained the same at 18 nm.


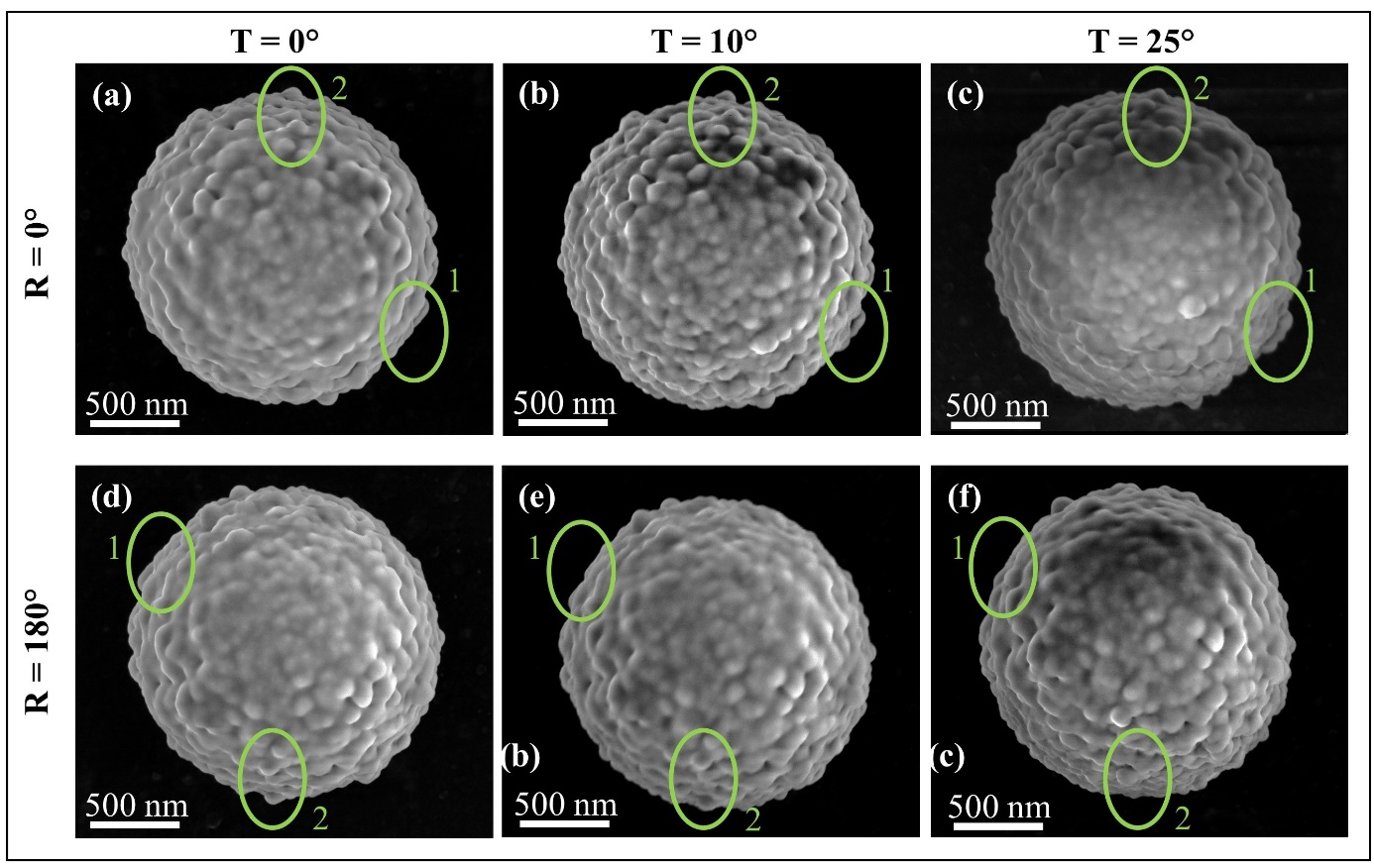


**Figure S1** SEM images of an induvial PS/Fe_3_O_4_/SiO_2_ core-shell-shell particle p_4_ recorded at 2 kV (a) without tilting and rotation, (b) with tilting at 10° without rotation, (c) with tilting at 25° without rotation, (d) without tilting at 180° rotation, (e) with tilting at 10°at 180° rotation, and (f) with tilting at 25°at 180° rotation (R: rotation degree, T: tilting degree).

Some of the large features seen in the SEM images begin to disappear both when we tilt the sample or repeat the tilting after rotation at 180°. This is valid for instance in case of the features of the contour shown by the green ellipse number 2. Although tilting the particle up to 25° did not change the obtained distances, repeating tilting after rotation resulted in lower distances in this region. Moreover, for some of the features, it was determined that the tilting or rotating procedure did not have a significant effect on the results obtained.


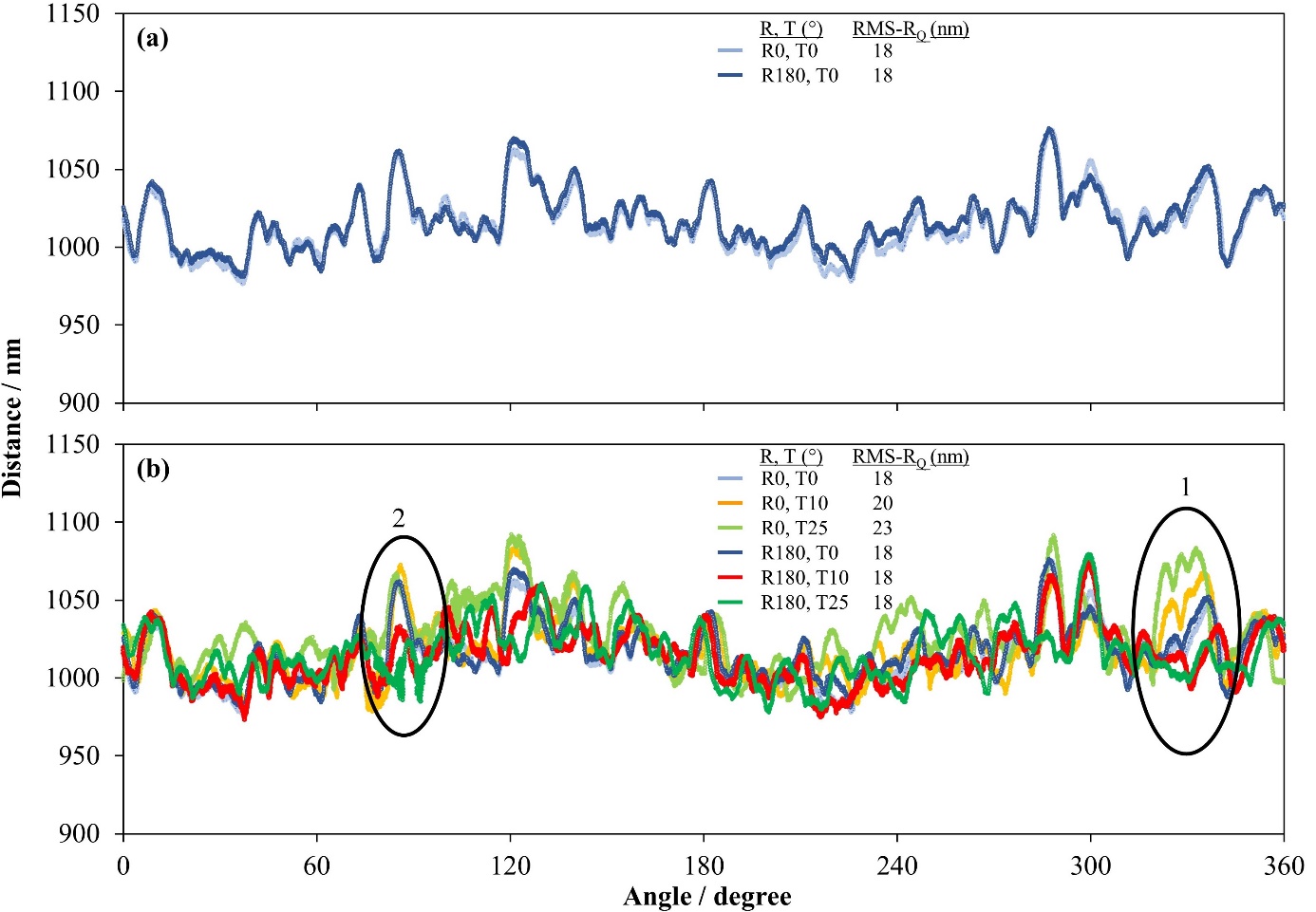


**Figure S2** Lateral profiles of the same PS/Fe_3_O_4_/SiO_2_ core-shell-shell particle p_4_ calculated from the SEM images from Figure S1: (a) comparison of profiles obtained without tilting at 0-degree rotation and 180-degree rotation, (b) comparison of profiles with tilting up to 25 degrees both with and without rotation at 180 degrees. Black ellipses number 1 and 2 correspond to the areas shown by green ellipses in Figure S1. (R: rotation degree, T: tilting degree).

## Scattering Properties in Flow Cytometry

Flow cytometric measurements were recorded with a BD Accuri C6 (for the details of the methodology see the Supporting Information Section 2.1 of our previous paper ^2^). The data were recorded with the sideward scattering channel (SSC) allowing for a comparison to the roughness values extracted from SEM images, as the SSC signals correlate with the structure and granularity of the particles. As shown in **Figure S3**, the two batches of particles exhibit different scattering distributions in the SSC channel. While the particles from batch 1 (**Figure S3**a) show a broader signal than pure polystyrene particles which, however, is clearly monomodal, batch 2 (**Figure S3**b) seems less monodisperse. The histogram of batch 2 is significantly broader and somewhat bimodal, supporting the finding of a second roughness cluster in SEM image analysis. This indicates a higher roughness for one population and more pronounced difference between the particles in the same batch.

The correlation of forward scattering (FSC), which is proportional to the size of the particles, and SCC can provide further information about the dispersity of the particle batches. As shown in **Figure S4**a batch 1 has a central population with one SSC-related roughness and size, i.e., the particles are clearly monomodal. **Figure S4**b shows the dispersity for batch 2, where two populations are clearly visible also in the dot plot representation; while the SSC-related roughness is distinctly different, the FSC-relates size is virtually identical.


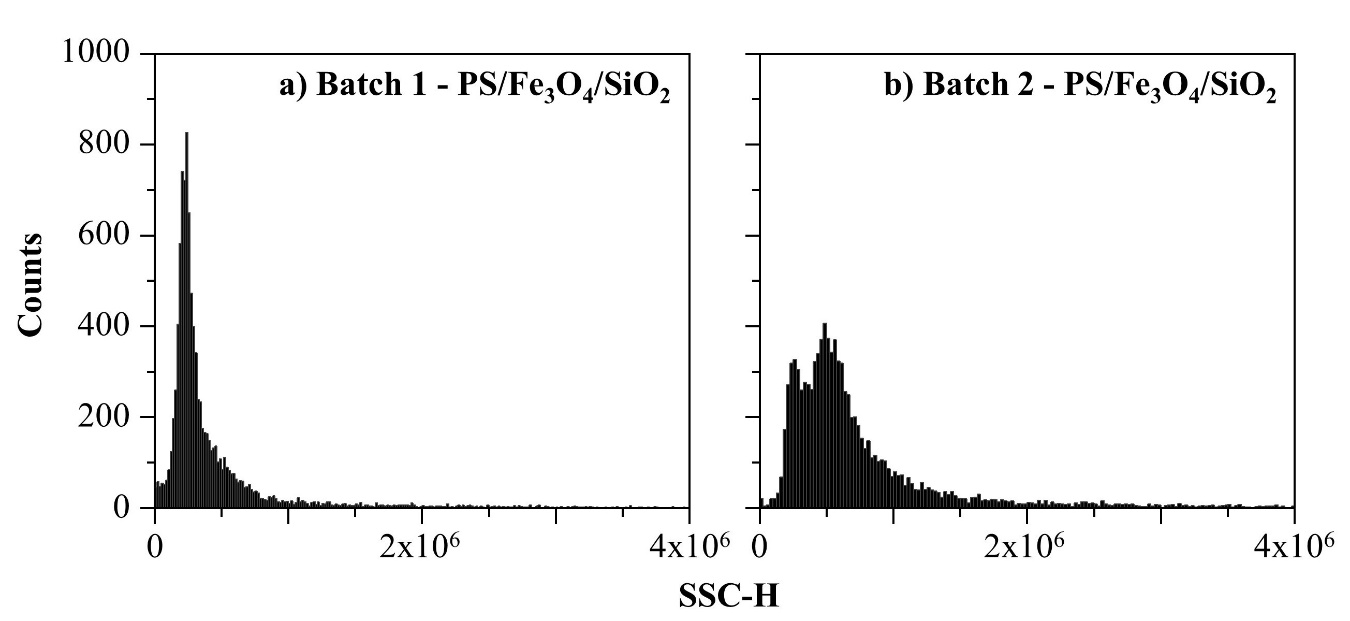


**Figure S3** Histogram of the shape parameter (SSC-H) for (a) PS/Fe_3_O_4_/SiO_2_ particles of batch 1 and (b) PS/Fe_3_O_4_/SiO_2_ particles of batch 2


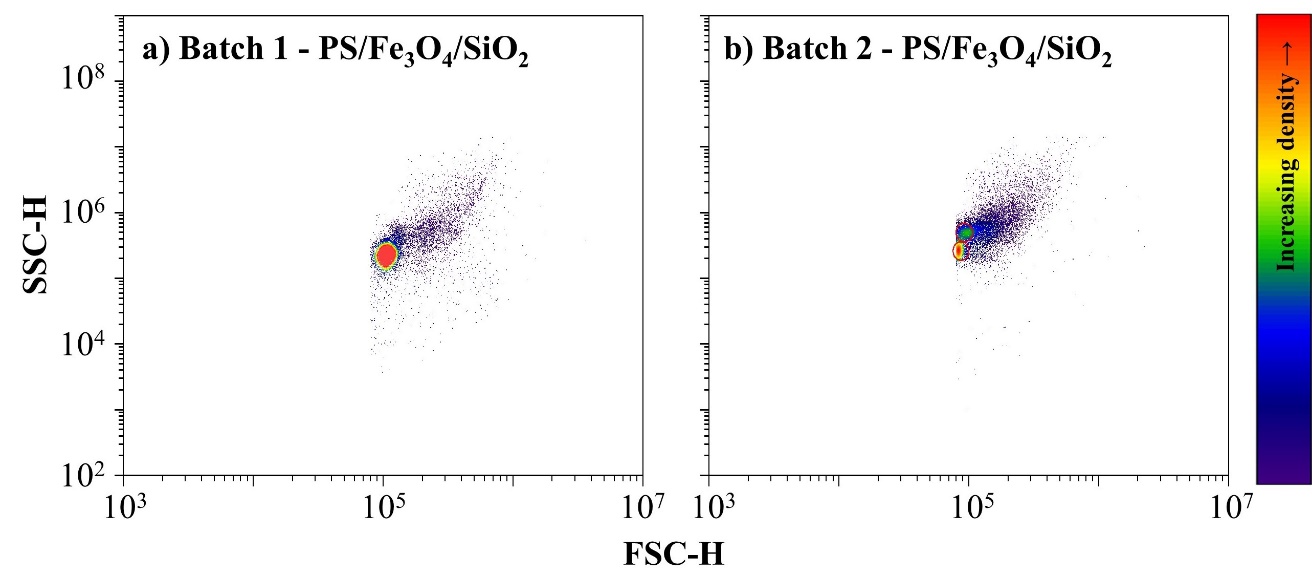


**Figure S4** Density scattering plot with size (FSC-H) and shape (SSC-H) parameters for (a) PS/Fe_3_O_4_/SiO_2_ particles of batch 1 and (b) PS/Fe_3_O_4_/SiO_2_ particles of batch 2

**References**

1 Tobias, C., Climent, E., Gawlitza, K. & Rurack, K. Polystyrene microparticles with convergently grown mesoporous silica shells as a promising tool for multiplexed bioanalytical assays. *ACS Appl. Mater. Interfaces* **13**, 207, <https://dx.doi.org/10.1021/acsami.0c17940> (2020).

2 Hülagü, D. *et al.* Generalized analysis approach of the profile roughness by electron microscopy with the example of hierarchically grown polystyrene–iron oxide–silica core–shell–shell particles. *Adv. Eng. Mater.* **24**, 2101344, <https://doi.org/10.1002/adem.202101344> (2022).
